# Fusobacterium nucleatum-Induced Empyema Masquerading as Lung Cancer: A Diagnostic Challenge

**DOI:** 10.7759/cureus.75003

**Published:** 2024-12-02

**Authors:** Ahmed R Fadel, Yasser N Ahmed

**Affiliations:** 1 Respiratory Medicine, Dartford and Gravesham NHS Trust, Dartford, GBR

**Keywords:** empyema, fusobacterium nucleatum, locus minoris resistentiae, oral-type pleural infection, pleural effusion, trapped lung

## Abstract

A 73-year-old man, an ex-smoker with a history of asbestos exposure and hypertension, presented with progressive shortness of breath, weight loss, loss of appetite, and fatigue. He was referred by a general practitioner for evaluation of a round lesion in the right lung. Chest computed tomography (CT) revealed a pleural-based mass and small right-sided pleural effusion that was not amenable to aspiration. Routine blood investigations revealed elevated levels of the inflammatory markers. He was discharged with a course of oral antibiotics, levofloxacin, and scheduled for a two-week follow-up on the lung cancer pathway.

The patient returned with worsening shortness of breath and enlarged pleural effusion. Ultrasound-guided diagnostic aspiration yielded purulent fluid, with cultures identifying *Fusobacterium nucleatum*. Cytological examination of the pleural fluid yielded negative results for malignant cells. Further investigations ruled out septic thrombophlebitis of the internal jugular vein (Lemierre syndrome) and septic mediastinitis. After excluding all other possible infection sites and conducting an oral examination, the pleural infection was attributed to periodontal disease. Empyema was effectively managed with antibiotics and drainage. However, empyema resulted in a visceral pleural rind, leading to an unexpandable lung. Surgical intervention was not pursued because of the patient’s comorbidities and clinical and biochemical resolution of the infection. This case highlights the importance of considering rare pathogens, such as *Fusobacterium nucleatum*, in pleural infections, particularly when linked to oral sources.

## Introduction

*Fusobacterium *species, particularly *Fusobacterium nucleatum*, are gram-negative anaerobes commonly found in the oral cavity. These organisms can act as opportunistic pathogens, causing invasive infections such as septic thrombophlebitis of the internal jugular vein (Lemierre syndrome) [1], septic mediastinitis, empyema, lung abscesses, and necrotizing pneumonia. Oral-type pleural infections (OPIs) involving *Fusobacterium nucleatum* are distinct from non-oral pleural infections (non-OPIs) due to their unique pathogenesis and microbial profile. The concept of locus minoris resistentiae [2] explains how areas of reduced resistance, such as ischemic or traumatized tissues, become predisposed to infection. Risk factors, including advanced age, male sex, and comorbidities such as alcohol abuse, increase susceptibility to these infections, particularly when hematogenous spread occurs from an oral source, such as periodontal disease.

This case highlights a diagnostic and therapeutic challenge: Oral-type pleural infections, such as *Fusobacterium nucleatum*-associated empyema, can mimic malignancy due to radiological findings of pleural masses and nodules. Early recognition, accurate diagnosis, and tailored management are crucial to improving the outcomes of such complex infections. Additionally, this report underscores the importance of using blood culture bottles for pleural fluid sampling to enhance the detection of anaerobic organisms [3], ultimately guiding effective treatment strategies, particularly for frail or nonsurgical candidates.

## Case presentation

**Figure 1:**
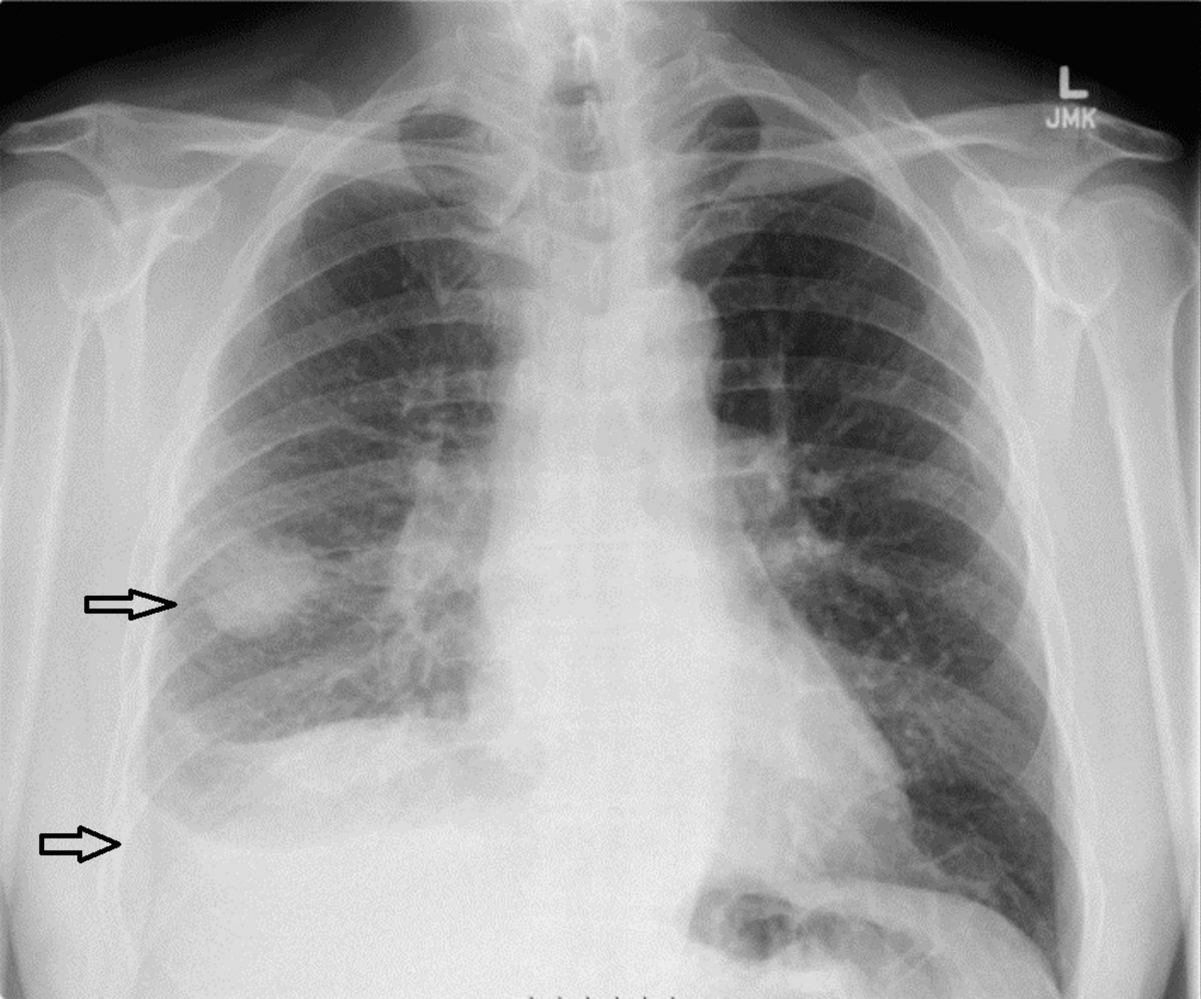
The chest X-ray revealed a 34 mm lesion in the right lung and small right-sided pleural effusion.

A 73-year-old man with a history of hypertension, extensive periodontal disease, and a fall on the right side of his chest three months earlier presented to our hospital after being referred by his general practitioner. The patient did not seek medical attention at the time of the fall, considering it a trivial injury. He reported a two-week history of increased fatigue, feeling unwell, and a productive cough with greenish sputum but denied fever. His symptoms had initially started two months earlier with decreased appetite, weight loss, and mild hemoptysis. He was referred to our hospital because of a lack of response to the initial antibiotics prescribed by his general practitioner and because of concerns about possible malignancy. Initial chest radiography revealed a 34 mm lesion in the right lung (Figure 1).

**Figure 2:**
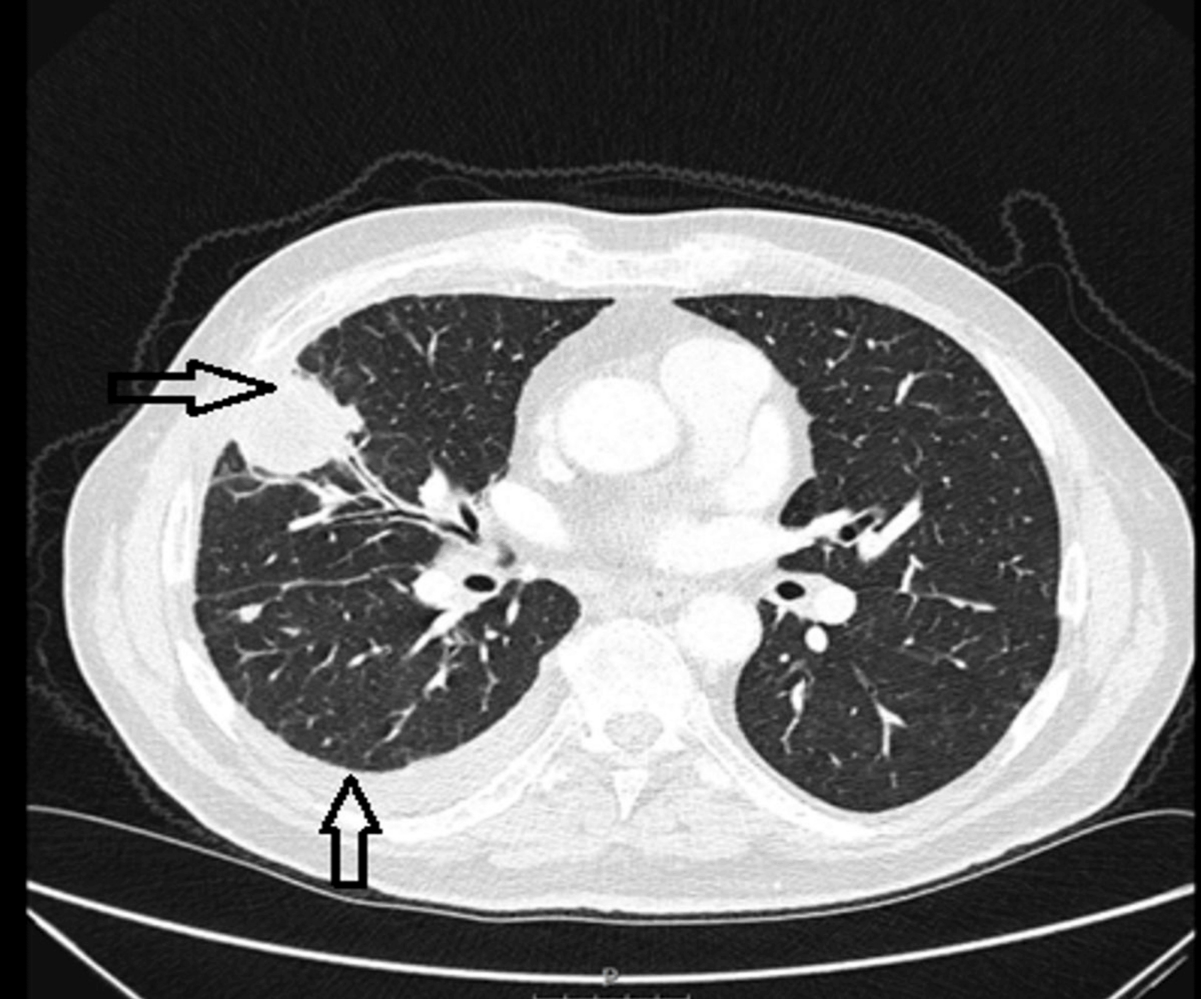
The CT scan of the chest's lung window showed a pleural-based, low-density mass in the periphery of the middle lobe, measuring 38 × 36 mm, and small right-sided pleural effusion.

**Figure 3:**
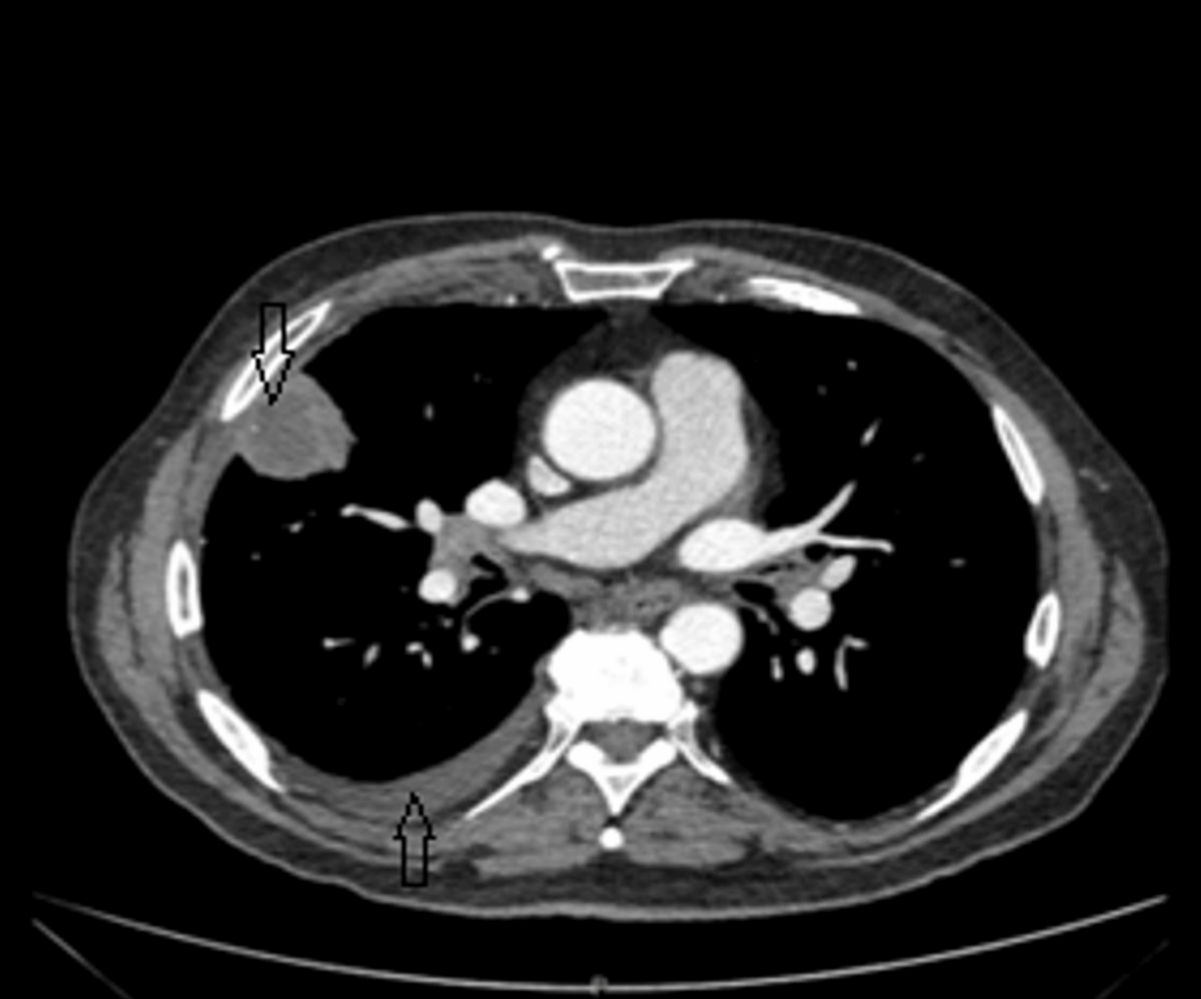
The CT scan of the chest's mediastinal window showed a pleural-based, low-density mass in the periphery of the middle lobe, measuring 38 × 36 mm, and small right-sided pleural effusion.

Subsequently, a CT scan of the chest, abdomen, and pelvis was performed to investigate possible malignancy. The CT revealed a pleural-based, low-density mass located in the periphery of the middle lobe, measuring 38 × 36 mm. The mass abutted the fissure and extended inferiorly, with an additional 9 mm nodule identified nearby. A small right-sided pleural effusion was also observed; however, diagnostic aspiration was not performed due to the minimal amount of fluid (Figures 2-3).

Blood investigations on admission revealed elevated infection markers, with a white blood cell (WBC) count of 8.8 × 10⁹/L (reference range: 4.0-11.0 × 10⁹/L) and a C-reactive protein (CRP) level of 97 mg/L (reference range: <5 mg/L). The patient was discharged with a course of oral levofloxacin and scheduled for review under the two-week cancer pathway.

**Figure 4:**
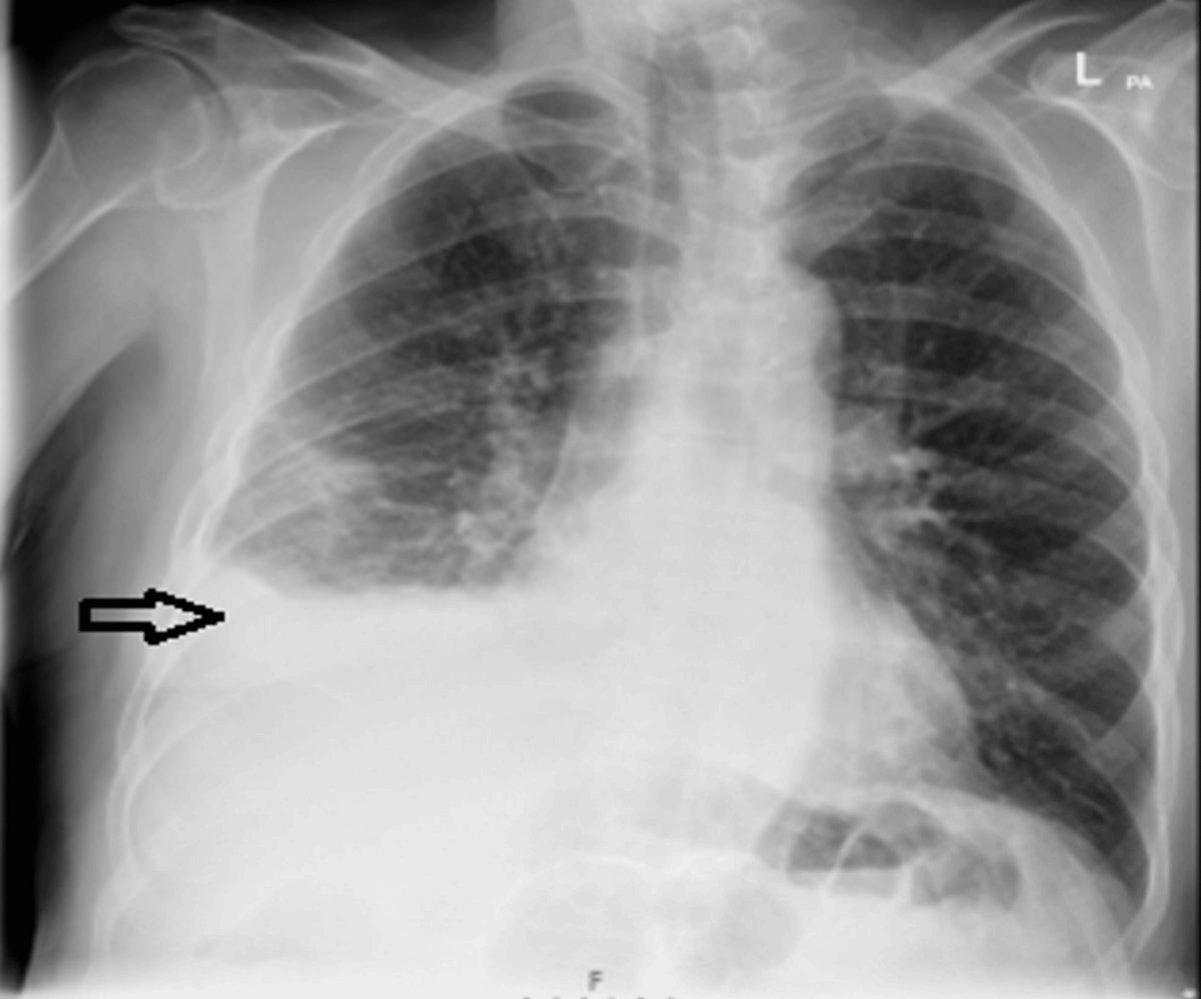
The chest X-ray showed increased consolidations in the right lower and middle lung zones, as well as increased right-sided pleural effusion.

A week later, he returned to the hospital with worsening chest pain and shortness of breath. A repeat chest radiograph showed increased consolidations in the right lower and middle lung zones, along with an increase in the size of the right-sided pleural effusion (Figure 4).

**Figure 5:**
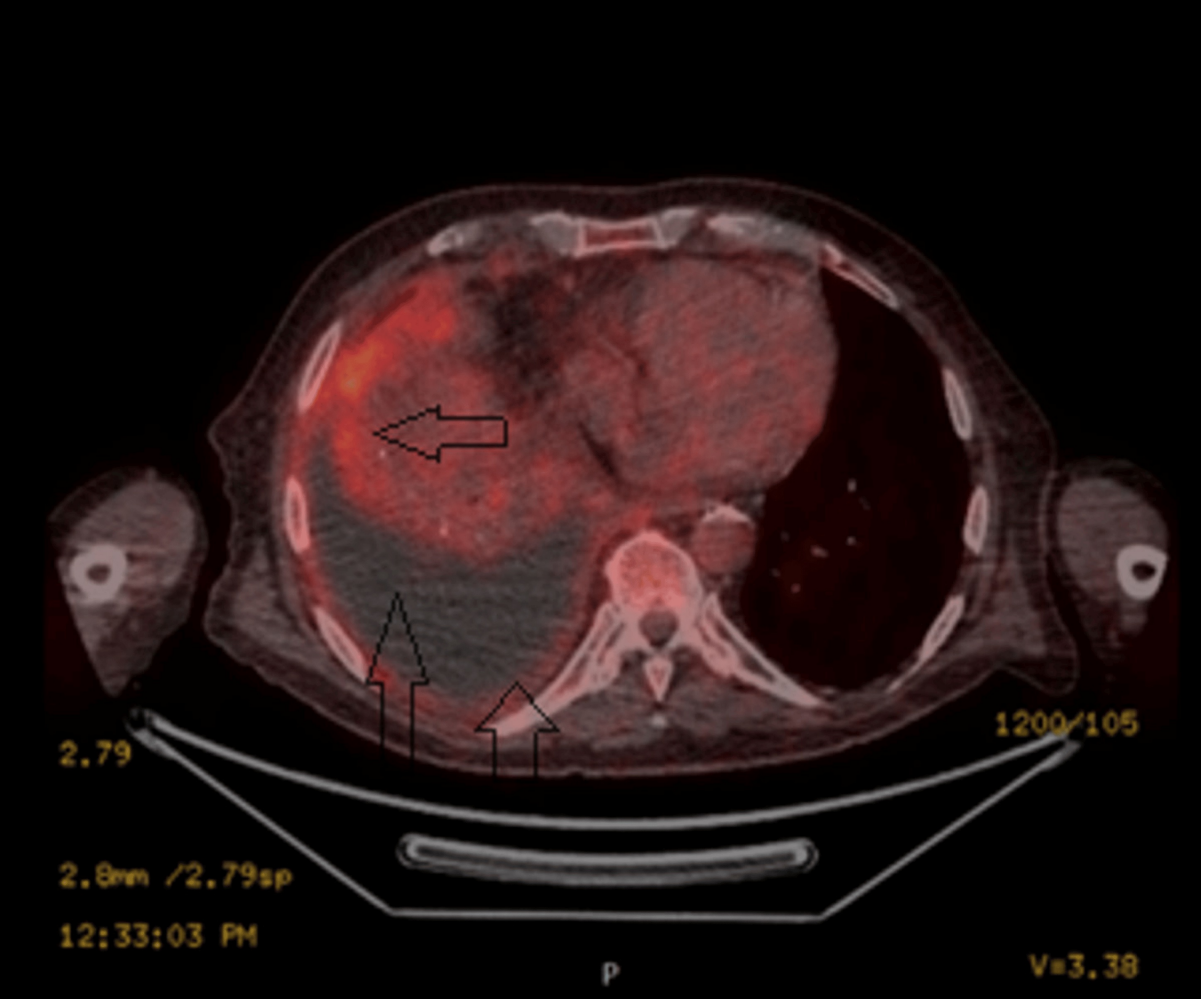
The PET scan showed a complete collapse of the right middle and lower lung lobes, along with a large right-sided pleural collection with moderately avid pleural uptake.

An urgent positron emission tomography (PET) scan showed a complete collapse of the right middle and lower lung lobes, along with a large right-sided pleural collection with moderately avid pleural uptake. The appearances were concerning for empyema, given the rapid progression and the presence of internal gas locules; however, malignancy could not be excluded (Figure 5).

**Figure 6:**
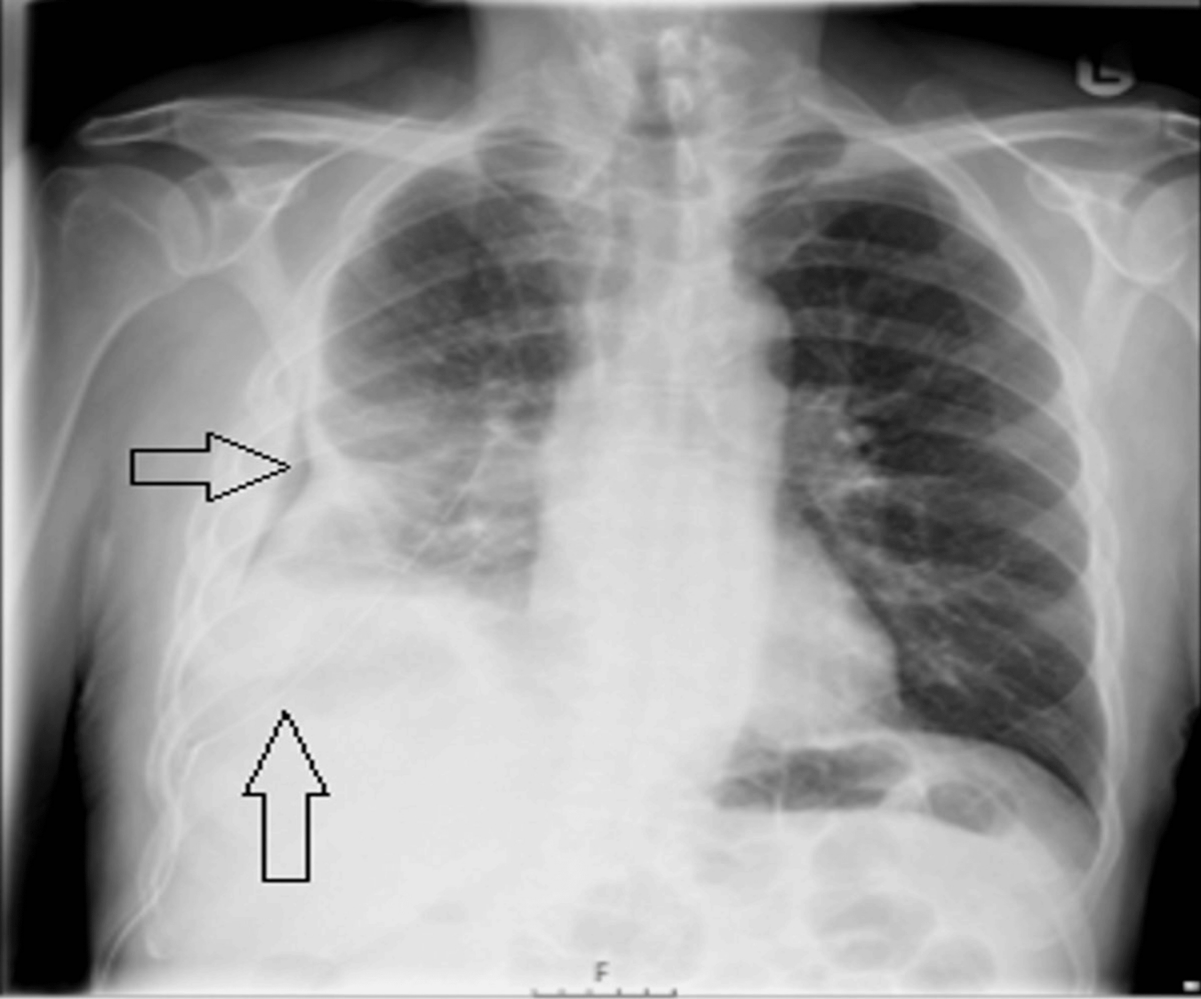
The chest X-ray showed the chest drain in situ, with significant pleural thickening and pneumothorax ex vacuo attributed to a non-expandable lung due to extensive visceral pleural rind.

Repeat blood investigations revealed a further rise in infection markers, with a CRP level of 184 mg/L (reference range: <5 mg/L) and a WBC count of 14.0 × 10⁹/L (reference range: 4.0-11.0 × 10⁹/L). Bedside thoracic ultrasonography revealed a heavily echogenic right-sided pleural effusion with multiple air loculi within the fluid (Video 1). Diagnostic aspiration drained foul-smelling pus, raising the suspicion of an anaerobic infection. Pleural fluid analysis revealed a protein level of 5.6 g/dL (serum ratio >0.5) and a lactate dehydrogenase (LDH) level of 8560 U/L (serum ratio >0.6), consistent with heavily exudative effusion. Subsequently, a chest drain was inserted, evacuating more than 1 liter of frank pus with an offensive odor. (Figure 6). To optimize microbial yield, pleural fluid samples were sent to blood culture bottles. Broad-spectrum antibiotics, including levofloxacin and metronidazole, were initiated, as advised by the microbiology team. Clinical improvement was observed following chest drain insertion and antibiotic therapy with a significant reduction in infection markers.

**Video 1:** Bedside thoracic ultrasound showed heavily echogenic right-sided pleural effusion, pleural thickening, and multiple air loculi suggesting likely pyopneumothorax.

**Figure 7:**
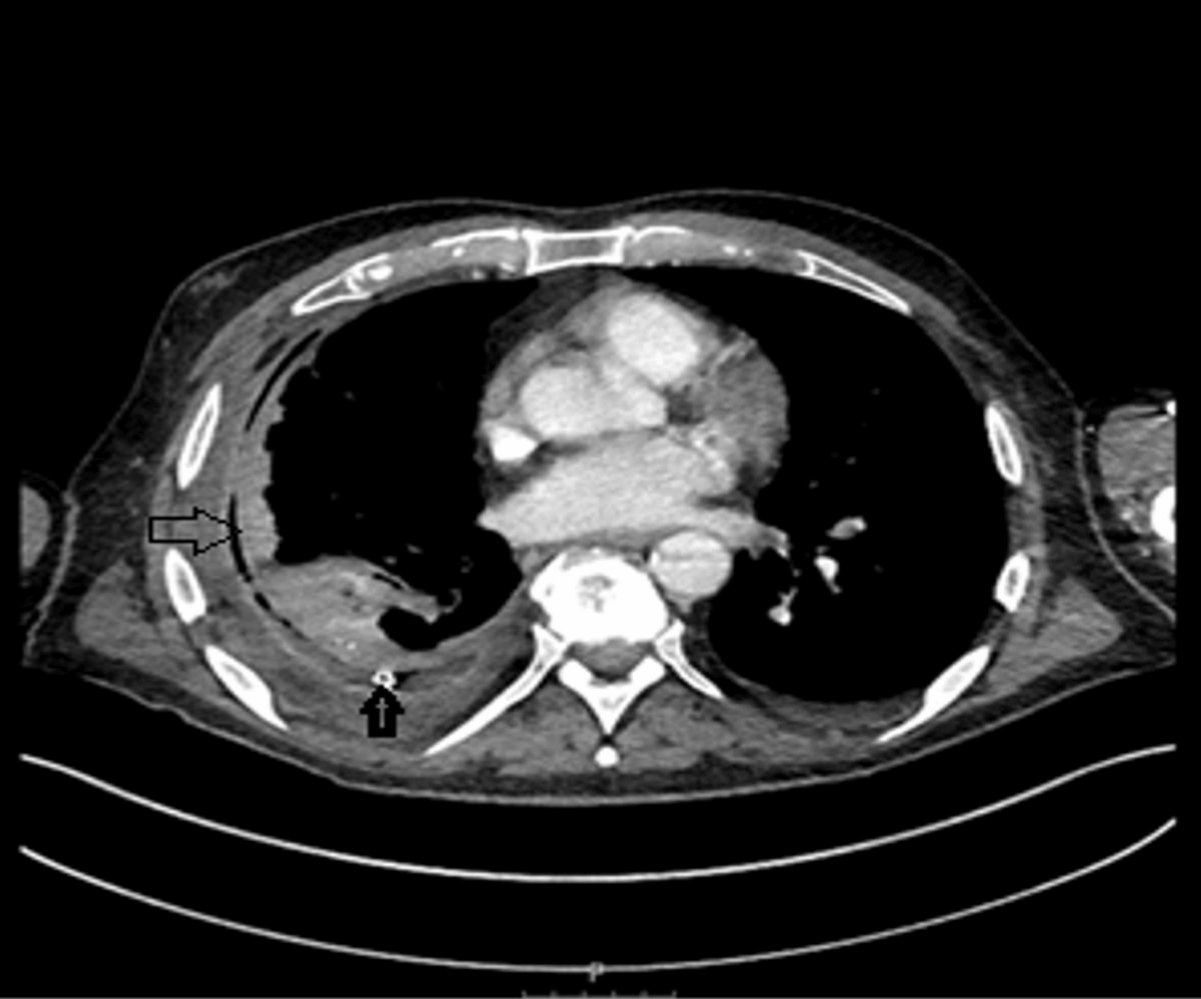
The CT scan of the chest with contrast revealed a chest drain in situ, a non-expandable lung due to a visceral pleural rind, and no evidence of mediastinal involvement.

**Figure 8:**
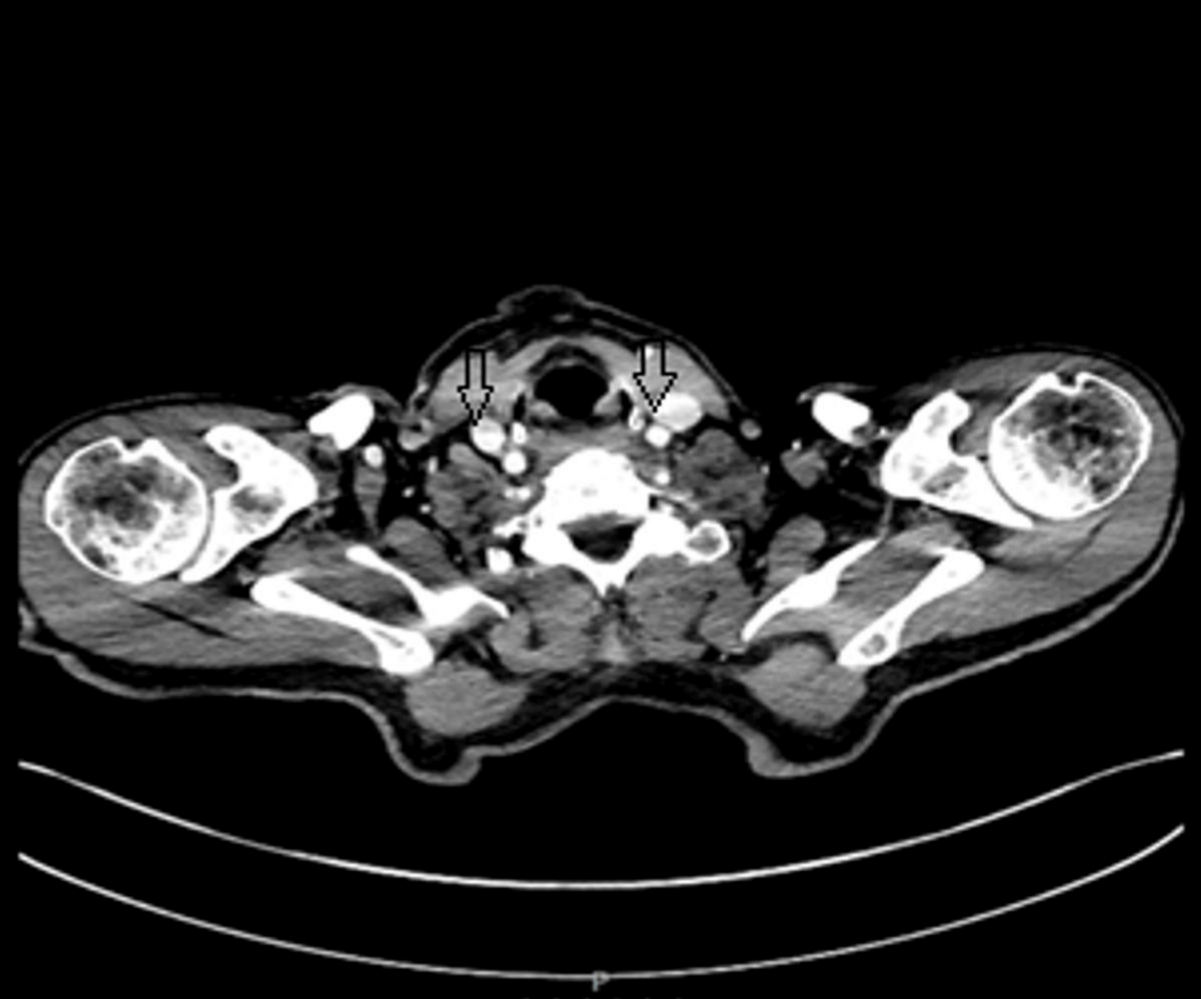
The CT scan of the neck with contrast revealed no evidence of internal jugular vein thrombus suggestive of Lemierre syndrome.

Pleural fluid cultures identified *Fusobacterium nucleatum* and *Leptotrichia *species, which are both gram-negative anaerobes. *Fusobacterium *species, in particular, are associated with unusual infections such as Lemierre syndrome, a condition characterized by septic thrombophlebitis of the internal jugular vein. To investigate a potential primary source, CT of the head, neck, and chest with intravenous contrast was performed, but no septic focus was identified (Figures 7-8).

A thorough examination revealed significant periodontal disease, and given the strong association of *Fusobacterium *species with such conditions, it was deemed the primary source of infection. The patient was referred to a dental team for outpatient evaluation and management.

Following cessation of drainage and notable clinical improvement, the patient was discharged with a six-week course of oral levofloxacin and metronidazole. This decision was guided by the presence of a non-expandable lung and a thick visceral pleural rind, coupled with the patient’s favorable response to conservative management. Surgical intervention was deemed unnecessary, particularly when considering comorbidities.

**Figure 9:**
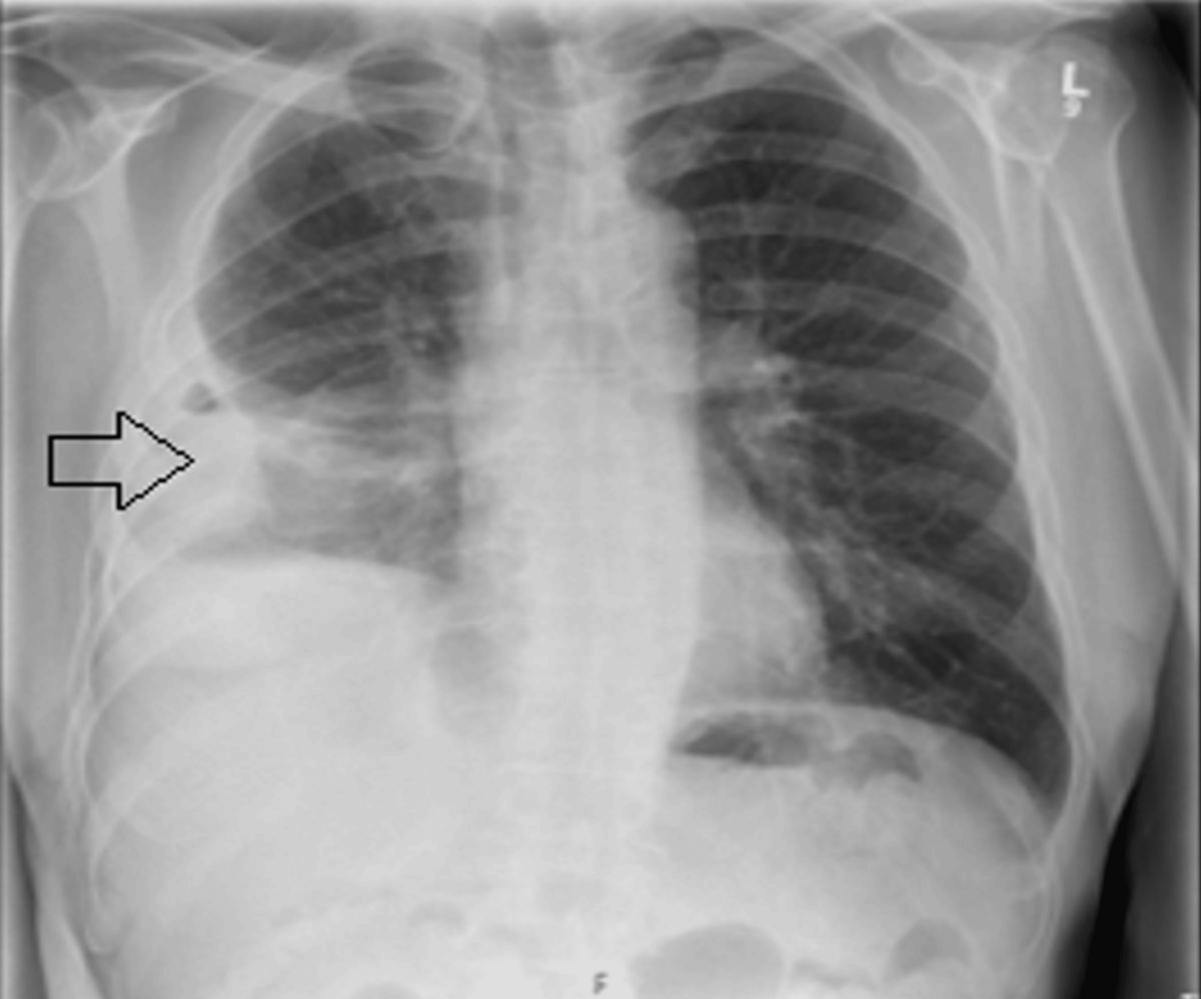
The chest X-ray revealed reaccumulation of small right-sided pleural effusion, likely secondary to trapped lung.

At the outpatient review one week later, the patient underwent a repeat chest radiography, which showed a reaccumulation of the right-sided pleural effusion (Figure 9). Diagnostic pleural aspiration revealed exudative effusion with a pH of 7.41, LDH of 670 U/L, and total protein level of 37 g/L. Pleural fluid was sent for culture in blood culture bottles, but no microbiological growth was observed. The cytological analysis was negative for malignant cells.

**Figure 10:**
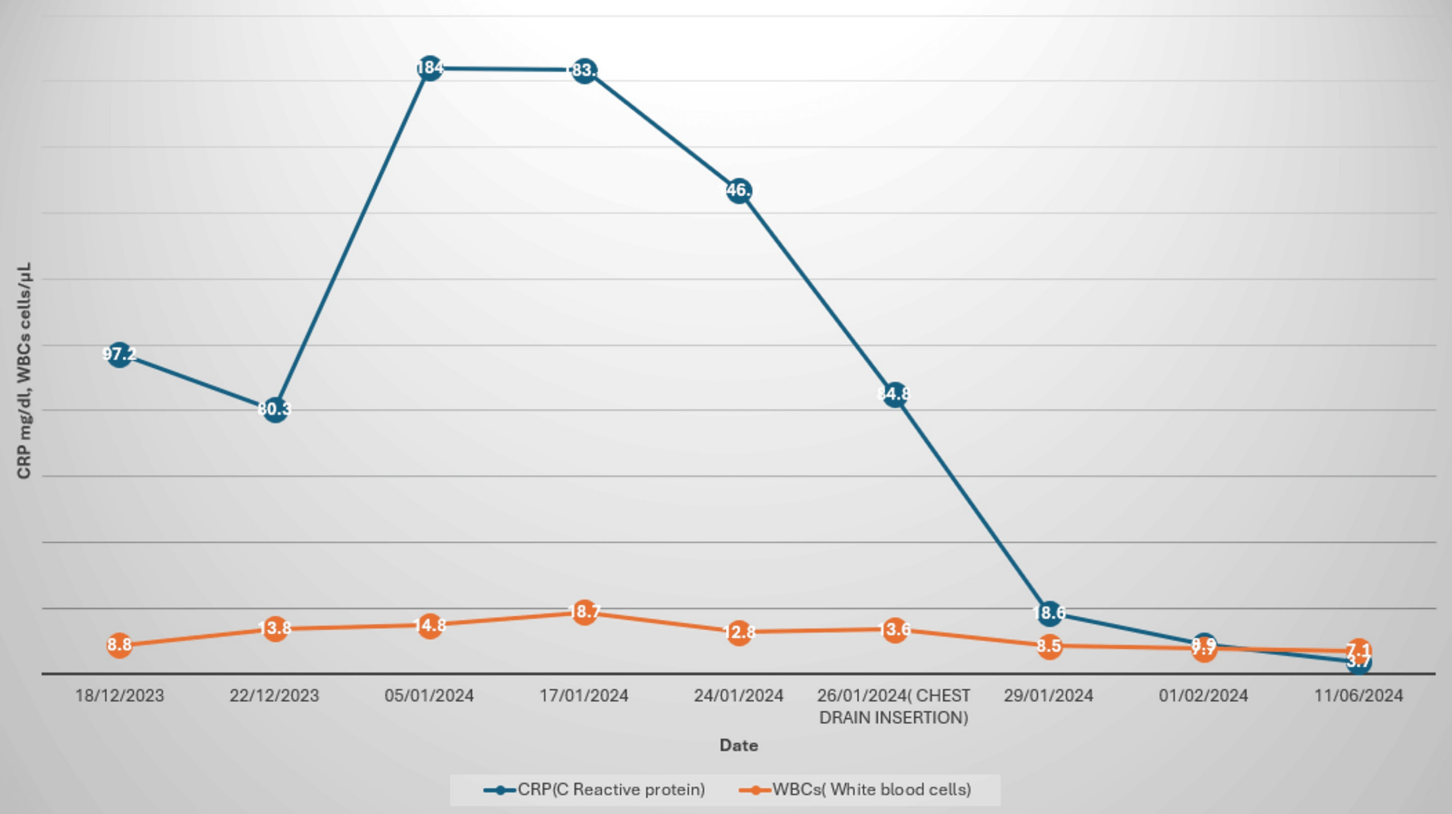
Trend of the inflammatory markers

The patient remained clinically stable, and his inflammatory markers remained low (Figure 10). He was referred to the surgical team, who determined that surgery was unnecessary due to clinical improvement and the high surgical risk. The reaccumulation of pleural effusion was attributed to a non-expandable lung caused by an extensive visceral pleural rind rather than a recurrence of the empyema.

## Discussion

Empyema is defined as the presence of frank pus in the pleural space or the growth of microorganisms in pleural fluid cultures. It is associated with significant morbidity, with a 30-day mortality rate of approximately 10.5%. The combined annual incidence of empyema in the United Kingdom and the United States is estimated at 80,000 cases [4].

**Figure 11:**
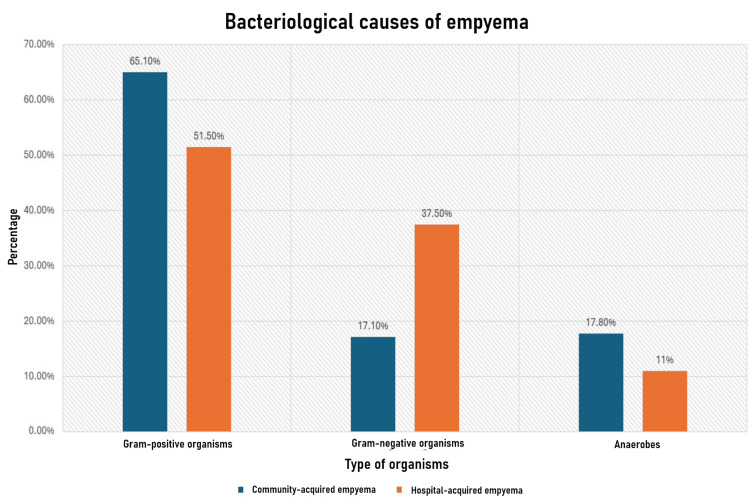
Bacteriological causes of empyema Adapted from [4-6].

Pleural fluid culture remains the most commonly used method for identifying causative organisms in empyema. However, the microbiological causes of empyema are highly variable and depend on several factors (Figure 11) [5].

However, this may underrepresent the role of anaerobes in empyema development. A Bulgarian study utilized the Crystal anaerobes identification system, the API System rapid ID 32 A, and/or routine methods and found that anaerobes were isolated in 72.4% of 198 patients. This higher isolation rate can be attributed to the sensitivity of anaerobes to aerobic conditions and the use of more advanced isolation methods, which are crucial in accurately identifying anaerobic organisms [7].

The most common isolates in this study were gram-positive anaerobes, accounting for 35.4% of the total, followed by *Fusobacterium *species, accounting for 34.7% of the isolates. *Fusobacterium necrophorum* was the most prevalent, constituting 27.2% of the total isolates [7].

*Fusobacterium *is a genus of strictly anaerobic, non-spore-forming, gram-negative bacilli comprising 13 distinct species according to modern taxonomy [8]. *Fusobacterium necrophorum* subspecies *funduliforme *and *Fusobacterium nucleatum* are the primary pathogens responsible for most invasive human infections [8]. Historically, *Fusobacterium *spp. have been strongly associated with Lemierre syndrome, a form of septic thrombophlebitis of internal jugular veins that typically arises as a complication of head and neck infections, most commonly tonsillitis. A systematic review found that Lemierre syndrome is predominantly linked to *Fusobacterium necrophorum*, whereas *Fusobacterium nucleatum* accounts for only 3% of cases [9].

**Figure 12:**
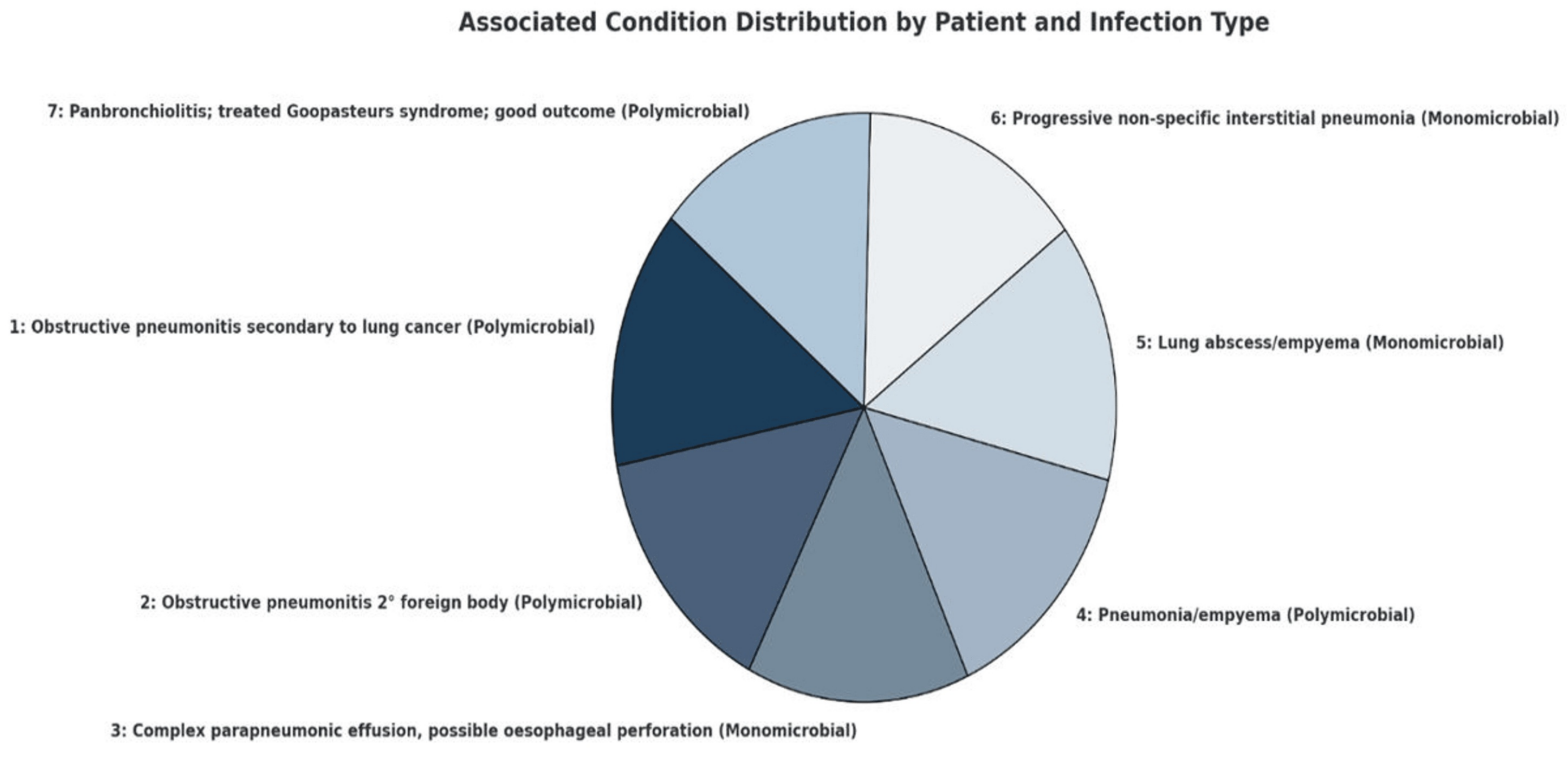
Clinical condition associated with Fusobacterium pleuro/pulmonary infections Adapted from [9].

Fusobacterial infections have been categorized into three distinct groups. The first group consisted of younger patients with fewer comorbidities, who primarily experienced head and neck infections. The second group included older individuals with multiple comorbidities who presented with soft tissue infections outside the head and neck region. The third group typically involves older patients, often with malignancies, particularly hematological malignancies, who present with *Fusobacterium bacteremia* without concurrent soft tissue or head and neck infections (Figure 12) [9].

*Fusobacterium nucleatum* is strongly associated with extensive periodontal disease, as observed in this patient. It is abundant in the oral cavities of both healthy and diseased individuals. The virulence of *Fusobacterium nucleatum* is linked to its ability to colonize and invade tissues, induce inflammation, and contribute to tumorigenesis [10].

Extraoral infections caused by *Fusobacterium nucleatum* are hypothesized to result from the expression of the surface adhesin protein, FadA. This protein increases vascular permeability and facilitates the penetration of *Fusobacterium nucleatum* into endothelial cells by binding to vascular endothelial cadherin (VE-cadherin) [10].

*Fusobacterium nucleatum* is associated with various non-infectious systemic diseases, including atherosclerotic cardiovascular disease [8], inflammatory bowel disease [10], rheumatoid arthritis [10-11], oral squamous cell carcinoma [10-12,13], gastrointestinal cancers, Alzheimer's disease, and breast carcinoma [10]. However, it is difficult to determine whether these relationships represent a causal link or are merely associations. Factors such as poor oral hygiene and smoking act as potent correlators of the social determinants of health, which may contribute to systemic inflammation and immune dysregulation, indirectly increasing susceptibility to these conditions. For example, while *Fusobacterium nucleatum* has been implicated in the pathogenesis of some cancers and inflammatory diseases, more robust evidence is required to establish direct causation. This highlights the importance of viewing oral health and smoking cessation as potential modifiable risk factors in the broader context of systemic health.

*Leptotrichia *species are gram-negative rods commonly found in the oral cavity, intestines, and genital tract. In this case, the isolation of *Leptotrichia *supported the suspicion that periodontal disease was the source of infection. *Leptotrichia *have been implicated in gingivitis, refractory periodontitis, abscesses, and endocarditis. It has also been isolated from the blood cultures of patients with malignancy and HIV [14].

**Figure 13:**
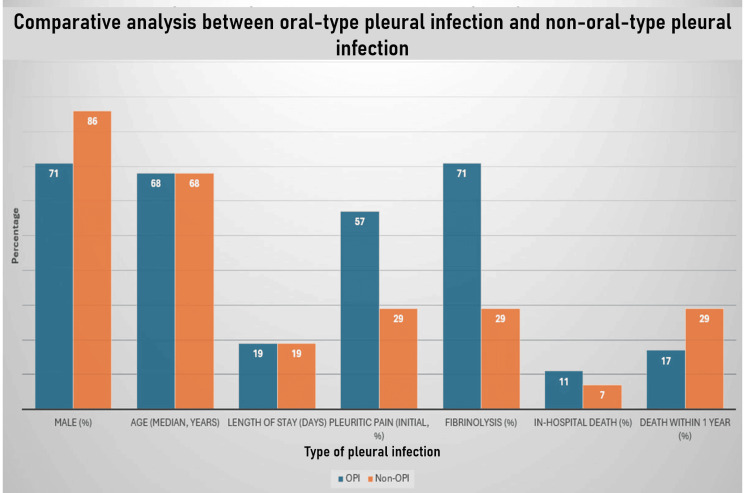
Comparative analysis between oral-type pleural infection (OPI) and non-oral-type pleural infection (non-OPI)

Oral-type pleural infection is a distinct clinical entity most commonly characterized by the isolation of *Streptococcus intermedius* and *Fusobacterium nucleatum*. These bacteria are frequently associated with polymicrobial infections and are commonly found in apical dental abscesses. They also often demonstrate growth in blood cultures, underscoring their strong association with dental infections (Figure 13) [2].

An intriguing concept highlighted in this study is locus minoris resistentiae, which refers to areas of reduced resistance in the body. These regions, often ischemic or devitalized, can arise from blunt trauma and are more commonly observed in elderly individuals, immunocompromised individuals, and those with excessive alcohol consumption. Such areas are particularly susceptible to the hematogenous spread of organisms from an odontogenic focus, even in the absence of a direct local cause. Risk factors for empyema, particularly OPI, include advanced age, male sex, alcoholism, and intravenous drug use [2].

Chest CT findings revealed that lung abscesses were more prevalent in OPI cases (38%) than in non-OPI cases (21%), with necrotizing pneumonia occurring almost exclusively in OPI [2]. These findings support the concept of locus minoris resistentiae, in which weakened or compromised tissues are more susceptible to infection. This can render infections more resistant to conservative management, including systemic antibiotics and chest drain insertion. The increased complexity of empyema in OPI, including adhesions and necrotic tissue, further complicates the treatment.

Surgery remains a viable option for complicated empyema cases that fail to respond to systemic antibiotics or drainage. Procedures may include open thoracotomy, thoracoscopic surgical adhesiolysis, decortication, abscess drainage, or lobectomy in cases of necrotizing pneumonia.

However, many patients with OPI are elderly and frail, making them unsuitable candidates for surgical intervention. Alternative salvage therapies are critical in such cases. Intrapleural fibrinolysis, as detailed in the Second Multicentre Intrapleural Sepsis Trial (MIST-2) protocol [15], is one such treatment approach. Another option is intrapleural injection of antibiotics, which, although primarily reported in case studies, may be considered for patients who do not respond to other therapies and are not candidates for surgery [16].

Although the patient experienced reaccumulation of pleural effusion during follow-up, the fluid did not appear to be infectious. This conclusion is supported by persistently low infection marker levels and clinical improvement without requiring additional drainage. The effusion was most likely attributed to trapped lung physiology caused by extensive visceral pleural rind [17].

## Conclusions

This case highlights the diagnostic and therapeutic complexities of oral-type pleural infections, particularly those involving rare pathogens, such as *Fusobacterium nucleatum* and *Leptotrichia *species. The patient presented with clinical and radiological findings that initially raised concerns regarding malignancy, underscoring the importance of considering alternative diagnoses, especially in the presence of risk factors such as periodontal disease. The identification of *Fusobacterium nucleatum* as the causative organism, likely originating from extensive periodontal disease, emphasizes the significance of thorough clinical evaluation and microbiological analysis in guiding treatment decisions. The management approach, which included broad-spectrum antibiotics and chest drainage, proved effective without necessitating surgical intervention despite the patient’s advanced age and associated comorbidities.

This case also reinforces the relevance of locus minoris resistanae in the pathophysiology of pleural infections, particularly in older and frail individuals. The reaccumulation of non-infectious pleural fluid attributed to trapped lung physiology highlights the need for individualized patient care and consideration of less invasive salvage therapies, such as intrapleural fibrinolysis. Further research is warranted to refine treatment strategies and improve outcomes in patients with oral-type pleural infections, especially those who are not candidates for surgical intervention.
